# Sugarcoated isolation: evidence that social avoidance is linked to higher basal glucose levels and higher consumption of glucose

**DOI:** 10.3389/fpsyg.2015.00492

**Published:** 2015-04-23

**Authors:** Tsachi Ein-Dor, James A. Coan, Abira Reizer, Elizabeth B. Gross, Dana Dahan, Meredyth A. Wegener, Rafael Carel, Claude R. Cloninger, Ada H. Zohar

**Affiliations:** ^1^School of Psychology, Interdisciplinary Center Herzliya, Herzliya, Israel; ^2^University of Virginia, Charlottesville, VA, USA; ^3^Ariel University Center of Samaria, Ariel, Israel; ^4^Ruppin Academic Center, Ruppin, Israel; ^5^Machon Mor Medical Center, Haifa, Israel; ^6^Haifa University, Haifa, Israel; ^7^Washington University, St. Louis, MO, USA

**Keywords:** glucose, attachment, social support, metabolic resources, avoidance, stress, social baseline theory

## Abstract

**Objective:** The human brain adjusts its level of effort in coping with various life stressors as a partial function of perceived access to social resources. We examined whether people who avoid social ties maintain a higher fasting basal level of glucose in their bloodstream and consume more sugar-rich food, reflecting strategies to draw more on personal resources when threatened. **Methods:** In Study 1 (*N* = 60), we obtained fasting blood glucose and adult attachment orientations data. In Study 2 (*N* = 285), we collected measures of fasting blood glucose and adult attachment orientations from older adults of mixed gender, using a measure of attachment style different from Study 1. In Study 3 (*N* = 108), we examined the link between trait-like attachment avoidance, manipulation of an asocial state, and consumption of sugar-rich food. In Study 4 (*N* = 115), we examined whether manipulating the social network will moderate the effect of attachment avoidance on consumption of sugar-rich food. **Results:** In Study 1, fasting blood glucose levels corresponded with higher attachment avoidance scores after statistically adjusting for time of assessment and interpersonal anxiety. For Study 2, fasting blood glucose continued to correspond with higher adult attachment avoidance even after statistically adjusting for interpersonal anxiety, stress indices, age, gender, social support and body mass. In Study 3, people high in attachment avoidance consume more sugar-rich food, especially when reminded of asocial tendencies. Study 4 indicated that after facing a stressful task in the presence of others, avoidant people gather more sugar-rich food than more socially oriented people. **Conclusion:** Results are consistent with the suggestion that socially avoidant individuals upwardly adjust their basal glucose levels and consume more glucose-rich food with the expectation of increased personal effort because of limited access to social resources. Further investigation of this link is warranted.

## Introduction

When God looked upon man, he or she contended that, “It is not good for the man to be alone.” (Genesis, 2:18). Research indeed suggests that humans draw strength from combined efforts in coping with situational demands. Social bonding serves security provision functions (Mikulincer and Shaver, 2007) and regulates stress, negative affect and arousal (Bowlby, 1973; Beckes and Coan, 2011). Some have suggested that highly social individuals can manage demands on their energy more efficiently (Coan, 2008; Beckes and Coan, 2011). For example, other people can help care for offspring (Ehrenberg et al., 2001), assist when ill or injured (Townsend and Franks, 1995), share resources (Roger and De Boer, 2001), and contribute vigilance for potential threats (Davis, 2010; Ein-Dor et al., 2011b).

Nevertheless, some individuals tend to distrust others’ goodwill, strive to maintain independence, and often distance themselves from other people when dealing with threats and negative emotions. These people, often called “avoidantly attached” (Mikulincer and Shaver, 2007), tend to cope with threats by deemphasizing distress and vulnerability and by attempting to cope independently, without seeking others’ help (Fraley and Shaver, 1997). Because they do not share the cost of many of life’s metabolically expensive activities, they are likely to employ a trait-like strategy of increased preparedness for individual decision making, problem solving, threat vigilance, and even the *regulation* threat vigilance—a strategy that increases their personal “budget” for rapidly accessible metabolic resources (Beckes and Coan, 2011; Gross and Proffitt, 2013). Recently, Bales (2012) have found indications for such a strategy among male titi monkeys, and Henriksen et al. (2014) have found such a strategy among less socially connected people (i.e., those who score high on measures of loneliness). In the present research, we examined this premise by testing (1) whether people who tend to avoid social ties—high scorers on attachment avoidance—maintain in their blood a higher basal concentration of circulating glucose, the most easily accessible metabolic fuel of the human body and brain (Vannucci and Vannucci, 2000); and (2) whether attachment avoidance corresponds with greater consumption of sugar-containing food such as carbohydrates.

Attachment theory (Bowlby, 1973, 1980, 1982) proposes that human beings possess an innate psychobiological system (*the attachment behavioral system*) that motivates them to seek proximity to significant others (*attachment figures*) when they need protection from threats. Throughout a history of interactions with attachment figures, people develop trait-like attachment orientations that are relatively stable over time (Ainsworth et al., 1978; Mikulincer and Shaver, 2007). The pioneering work by Ainsworth et al. (1978) has indicated that when attachment figures regularly respond sensitively to a person’s needs, he or she develops a sense of attachment security while acquiring constructive strategies for coping with threats and regulating negative emotions. “Secure” people generally cope with threats either by relying on internal resources developed with the help of security-enhancing attachment figures or by effectively seeking support from others or collaborating with them (Shaver and Mikulincer, 2002). Secure individuals generally have high self-esteem, trust other people, and perceive the world as a relatively safe place (Mikulincer and Shaver, 2007). These socially oriented tendencies might also help them to reduce energy expenditure relative to energy consumption (Coan, 2008; Gross and Proffitt, 2013). For example, their vigilance-related processing might decrease because they can rely on the combined efforts of others to detect threats (Roberts, 1996). Indeed, secure individuals are less attentive to signs of danger, and show the longest delays in warning others about dangers they do detect (Ein-Dor et al., 2011a,b; Ein-Dor and Orgad, 2012). Secure people may also reduce their energy expenditure by depending more on others to identify and acquire resources, assist with health-related needs, and help nurture offspring (Coan, 2008; Gross and Proffitt, 2013).

Attachment theory suggests that when attachment figures are unavailable, unreliable, or rejecting of bids for support, a person may become chronically wary of depending on others (Bowlby, 1982; Mikulincer and Shaver, 2007). A common manifestation of insecure attachment is *avoidance*, which is marked by extreme independence, lack of intimacy and self-disclosure, and emotion-regulation strategies characterized by social distancing. Citing the brain’s tendency to act as a Bayesian predictor of future outcomes, Beckes and Coan (2011) contend that “a history of finding others to be relatively unhelpful and unreliable may lead to priors that predict low levels of social support, biasing individuals to make “bets” that social resources are unlikely to obtain, and leading to an increased recruitment of personal resources in the presence of various life challenges.” (p. 981). Because people who avoid social ties need to recruit their own resources more often than their secure counterparts, they might need more rapid access to freely available metabolic resources—a budgeting strategy that could, in theory, lead to greater basal levels of glucose in the bloodstream, and to greater consumption of sugar-containing food. Recently, Dickinson et al. (2014) have supported the notion that glucose intake relates to Bayesian-based decision making and not to heuristic-based ones. In the present research, we examine this possibility by relating attachment avoidance disposition with glucose budgeting strategies. Recently, Bales (2012) have revealed several indications for an equivalent mechanism among male titi monkeys. In the wild, male titi monkeys leave their family and travel for long distances looking for a mate. The researchers have employed a laboratory setting and discovered that after the male titi monkeys detach from their kin, they show an increase in blood glucose concentration, which decreases once they reattach with a mate. Regarding glucose intake, Henriksen et al. (2014) have indicated that perceived loneliness was linked with elevated intake of sugar-rich beverages, whereas indices of social connectedness, such as relationship satisfaction, being married, having supporting friends, and having a sense of togetherness at work, was related to lower intake of sugar-rich beverages. These tendencies were significant even after adjusting for body mass index, self-image, physical activity and mental health. Jaremka et al. (2015) also linked loneliness with higher hunger among women with low body mess.

Glucose is the predominant organic fuel for all animal species, including humans (Kety, 1950). This is equally true for all of the body’s activities, including cerebral metabolism (Heininger, 2002). Moreover, glucose tends to be efficiently utilized to produce the maximal amount of chemical energy possible (Vannucci and Vannucci, 2000). Importantly, circulating blood glucose levels are sensitive to negative emotions (Skaff et al., 2009), and can be conditioned to prevailing social circumstances over longer periods of time (Woods and Kuskosky, 1976; Brummett et al., 2005; Henriksen et al., 2014) via several central mechanisms (Anthony et al., 2006). Lower activity in at least one such mechanism, the ventral striatum, is associated with greater insulin resistance and higher circulating blood glucose (Ryan et al., 2012). Moreover, the ventral striatum tends to be less active among people who avoid social attachments (Vrtička et al., 2008). If people who tend to avoid social ties place their own personal resources under greater demand—including demands that can be unpredictable and urgent—then maintaining higher basal glucose levels via these and other central mechanisms could provide them with the metabolic resources to more rapidly and independently overcome life’s many challenges.

Research also suggests that various psychological processes influence the intake of sugar-containing food (e.g., Henriksen et al., 2014) and that depletion of body-energy elicits specific motivations and behaviors that allow the acquisition and replenish of metabolic resources (Gailliot and Baumeister, 2007; Gailliot et al., 2007; Wang and Dvorak, 2010; Aarøe and Petersen, 2013). For example, Wang and Dvorak (2010) have shown that increasing blood glucose levels by drinking sugar-rich beverage, led participants to place more value in future reward over present goods. Such shifts in motivations and behaviors may be interpretable as adaptive strategies to maintain optimal level of glucose in the blood (Aarøe and Petersen, 2013). Social baseline theory (Beckes and Coan, 2011; Gross and Proffitt, 2013; Coan and Sbarra, 2015), suggests that a person’s optimal level of glucose in the bloodstream may be set by the expected quality and breadth of his or her social network. People with asocial tendencies, such as those high in attachment avoidance, may need set this optimal level higher, because they expect others either cannot or will not aid them when in need. Along with other centrally mediated mechanisms, the maintenance of higher glucose levels may entail eating more sugar-rich food.

In the present research, we designed four studies that examined the link between attachment avoidance, basal glucose level, and consumption of sugar-rich food. In Study 1, participants simply completed a self-report measure of attachment avoidance and had their finger pricked to measure blood glucose concentration. We predicted that the higher the attachment avoidance, the greater the basal glucose level. In Study 2, we examined whether the association between avoidance from social ties and basal glucose would generalize to (a) a different culture (Israel as compared with United States); (b) women and men from a different age group (adults and late adults as compared with young adults); and (c) a different measure of attachment avoidance. In Study 2, we also adjusted the analysis for the possible contributions of body mass index, gender, age, social support, time of assessment and three robust indices of stress–self-report ratings on anxiety-related symptoms, physicians’ diagnosis of hypertension symptoms, and fasting basal ratio of cortisol to adrenal androgen dehydroepiandrosterone (DHEA). In Study 3, we examined the link between trait-like attachment avoidance, manipulation of an asocial state, and consumption of sugar-rich food. Study 3 allowed us to examine the causal link between asocial tendencies and glucose-related correlates. We predicted that the higher the attachment avoidance, the greater the consumption of sugar-rich food. We also predicted that under a state of asocial expectations, people would consume more sugar-rich food than under a control condition. In Study 4, we examined whether manipulating the social network will moderate the effect of attachment avoidance on consumption of sugar-rich food. Specifically, participants underwent a challenging task either alone or in pairs, and then ate cereals high in sugar for breakfast. We predicted that attachment avoidance will be linked with greater consumption of sugar-rich food specifically in the paired condition, because asocial people do not expect to benefit from the aid of others.

## Study 1

In Study 1, we examined whether attachment avoidance predicts fasting basal levels of glucose in the blood. To this end, participants completed self-report measure of attachment avoidance, and gave a blood sample to assess their blood glucose concentration.

### Materials and Methods

#### Participants

As part of a larger study of social support, 60 undergraduate women ranging in age from 18 to 21 years were recruited in pairs from the University of Virginia’s Department of Psychology Participant Pool. Participants were instructed to avoid all eating and drinking (except water) for 3 h prior to their participation, and were informed that participation involved, among other things, finger pricks for the purpose of measuring blood glucose concentration. All participants consented to the study, and were either given course credit or compensated financially for their participation. Study 1 was approved by the University of Virginia’s institutional review board (IRB; granted to JAC and MAW; HSR # 14240).

#### Materials and Procedure

Results reported here are drawn from a separate set of studies designed to investigate associations between emotion regulation and blood glucose concentration. Participants were separated upon arrival, and, after consenting to the study, gave a history of everything they had orally consumed for the past 12 h. If participants had adhered to the fasting procedure, they then gave a baseline blood glucose measure before engaging in additional study procedures not reported here. Blood glucose levels were measured with a Hemocue 201 Glucose tester (in mg/dL). At the end of the session, participants filled out a variety of questionnaires. Attachment orientations were assessed with the experiences in close relationships-revised scales (ECR-R; Fraley et al., 2000). Participants rated the extent to which each item was descriptive of their feelings in close relationships on a 7-point scale ranging from *not at all* (1) to *very much* (7). Eighteen items assessed attachment anxiety (e.g., “I am afraid that I will lose my partner’s love”) and 18 assessed avoidance (e.g., “I prefer not to show a partner how I feel deep down”). The reliability and validity of these scales have been repeatedly demonstrated (Fraley et al., 2000; Mikulincer and Shaver, 2007). In the present study, Cronbach αs were 0.89 for the anxiety items and 0.94 for the avoidance items, and the two scores were significantly correlated, *r*(58) = 0.37, *p* < 0.01. After completing these questionnaires, participants completed a socio-demographic questionnaire and were debriefed and thanked.

### Results and Discussion

**FIGURE 1:**
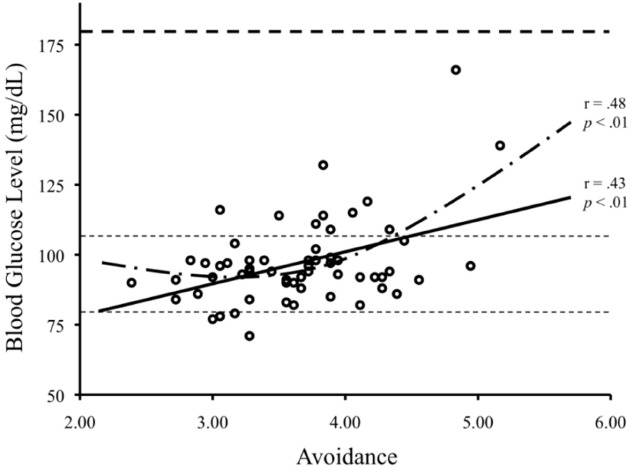
**The scatterplot depicts the association between attachment avoidance and fasting basal glucose level.** Consistent with our hypothesis, the higher the participants’ attachment avoidance score, the higher their fasting basal glucose level. Also, as the attachment avoidance score increased, the association between attachment avoidance and fasting basal glucose level increased in its relative magnitude. Note that the area within the light dashed lines represents the normal range of fasting basal glucose levels. By contrast, the heavy dashed line near the top depicts the level at which fasting basal glucose levels become clinically significant.

Participants’ fasting basal glucose level was examined using a curve estimation regression analysis (estimating linear and quadratic relations), in which participants’ attachment avoidance score served as the predictor, and their fasting basal glucose level served as the outcome measure. Estimating a linear association between attachment avoidance and fasting basal glucose level, we observed that the higher the participants’ attachment avoidance score, the greater their fasting basal glucose level, *F*(1, 58) = 12.89, β = 0.43, *R^2^* = 0.18, *p* < 0.001. Adding the quadratic estimation yield marginally significant increment in the association, *t*(57) = 1.96, *p* = 0.055, boosting the β to 0.48 and the *R^2^* to 0.23 (see Figure 1). When time of testing was included in the regression model, there was no effect of time of testing, *t*(57) = 1.24, *p* = 0.22, and the model estimating linear and quadratic effects of attachment avoidance on fasting basal glucose remained significant, *F*_(3,55)_ = 6.28, *p* = 0.001, *R*^2^ = 0.21. No similar associations were observed between fasting basal glucose and attachment anxiety.

In line with our prediction, women who tend to avoid depending on others for support had greater fasting basal levels of glucose in their blood than their more socially oriented counterparts. The higher levels of basal blood glucose found in highly avoidant individuals may serve as a metabolic reservoir that provides people high in attachment avoidance with the needed energy for rapid, independent responses to unpredictable contextual demands. Study 1, however, cannot rule out an alternative explanation that social avoidance promotes elevated levels of tension and stress and thus, in turn, higher levels of fasting basal blood glucose. In fact, among humans as well as many other animals, elevated basal blood glucose is a reliable marker of stress (Armario et al., 1996). Moreover, these results do not speak to whether the association between attachment avoidance and fasting basal blood glucose generalizes to men. We designed Study 2 to address these questions.

## Study 2

In study 2, we collected data on people’s attachment orientations (avoidance and anxiety), fasting basal blood glucose level, three independent indicators of tension and stress, and the following control measures: age, gender, social support, BMI, and time of assessment. Next, we examined whether attachment avoidance was positively related to fasting basal glucose levels independent of our tension and stress measures. We selected multiple methods to measure tension and stress, including self-report ratings on anxiety-related symptoms, physicians’ diagnosis of hypertension symptoms, and fasting basal ratio of cortisol to adrenal androgen DHEA. Elevated cortisol/DEHA ratios are associated with tension, stress, and related psychopathology (Goodyer et al., 2001). Following Wang and Dvorak (2010), we also adjusted the analysis for the possible effects of body mass index, gender, age, social support, and time of assessment.

Also, to address the generalizability of the present research, we sampled 285 Israeli adults (49.82% men) who were significantly older than the participants in Study 1, and used a different measure of attachment orientation. Thus, we were able to examine whether the results of Study 1 could be replicated in a sample that was (a) from a different culture, (b) mixed by gender, (c) older, and (d) assessed using a different measure of attachment avoidance. We hypothesized that attachment avoidance would be associated with higher fasting basal levels of glucose, and that the indicators of tension and stress would not account for that association.

### Materials and Methods

#### Participants

Study 2 was part of ongoing longitudinal research conducted at Ruppin Academic Center (Cloninger and Zohar, 2011). Two-hundred-eighty-five Israeli participants (143 women and 142 men), ranging in age from 42 to 90 years (*Mdn* = 58), volunteered to participate in the study, which included a free medical examination at a well-known medical facility (Mor Institute for Medical Data Ltd). Study 2 was approved by the Hillel Yaffe Medical Center’s Helsinki committee (granted to RC; HSR # 42\2007).

#### Measures and Procedure

The study spanned two sessions. In the first session, participants, who were recruited by a series of public lectures, mailbox pamphlets, and word of mouth, were individually invited to Ruppin Academic Center for a morning of interview, self-report, and cognitive testing. Attachment orientations were assessed with a Hebrew-language questionnaire developed by (Mikulincer et al., 1990; Mikulincer and Erev, 1991). In completing this questionnaire, participants rated the extent to which each item was descriptive of their ECR on a 7-point scale ranging from *not at all* (1) to *very much* (7). Eight items assessed avoidant attachment (e.g., “I am uncomfortable when other people get too close to me”) and 7 assessed anxious attachment (e.g., “I worry about being abandoned”). In the present study, Cronbach αs were 0.67 for the anxiety items and 0.76 for the avoidance items. Previous research has shown high concordance between this brief measure and the 36-item ECR measure (Brennan et al., 1998). Mean scores were computed for each scale, and the two scores were significantly correlated, *r*(280) = 0.55, *p* < 0.001.

General anxiety level was assessed with a Hebrew version of the Brief Symptom Inventory (BSI; Derogatis and Melisaratos, 1983)—a 53-item self-report inventory in which participants rate the extent to which they have been aggravated (0 = “not at all” to 4 = “extremely”) in the past week by various symptoms. The BSI anxiety subscale comprised six items (e.g., “Feeling tense or keyed up”), and its reliability and validity have been repeatedly demonstrated (Boulet and Boss, 1991). In the present study, Cronbach α was 0.81, and thus self-rated anxiety was calculated by averaging the item ratings.

Social support was assessed with the multidimensional scale of perceived social support (MSPSS; Zimet et al., 1990). It includes items to assess perceived support from friends, family and an intimate partner (e.g., “There is a special person who is around when I am in need”). In the present study, Cronbach α was 0.93, and thus a total score of perceived social support was calculated by averaging the item ratings.

Upon completion of the first session, participants were given a referral for a medical examination at the Mor Institute for Medical Data Ltd, and the medical center administrator was given the contact details of the prospective patient. Participants were invited to the medical center in the morning after a fast of 12 h. We controlled for time of awakening, morning activity, caffeine consumption and smoking, factors that can affect morning cortisol levels. All participants were instructed not to exercise before coming to the examination. The medical examination was conducted independently of all other study variables, by medical staff blind to the study goals and hypotheses. Upon participants’ arrival, a nurse drew blood samples into serum tubes containing aprotinin (500 kallikrein-inhibiting units, or KIU, per ml of blood). The samples were centrifuged at 1,600 × g for 15 min at 4°C, and then transferred to plastic tubes and stored at –80°C. Cortisol was measured by the TKCO1-Coat-A-Count kit (Diagnostic Products Corporation, Los Angeles, CA, USA), and DHEA was assessed with the DHEA-DSL-9000-Active^TM^ DHEA coated tube radioimmunoassay kit (Diagnostic System Laboratories, Webster, TX, USA). Basal glucose levels were measured with the Roche Diagnostics Serum Work Area Modular Analytics P-800 auto-analyzer (Roche Diagnostics, Basel, Switzerland).

After the blood tests, an expert physician examined the participants to establish the occurrence of various medical conditions, including clinical hypertension, diabetes, and obesity. Test results were sent to the last author’s (AHZ) research laboratory as well as to the participants. Upon request, the results were also sent to the participants’ general practitioner in the community.

### Results and Discussion

Participants’ fasting basal glucose level was examined using hierarchical regression^1^. In the first step of the analysis, we introduced participant age as a predictor because age alone can cause systematic variations in fasting basal glucose levels (Shimokata et al., 1991). Following Wang and Dvorak (2010), we also adjusted the analysis for the possible effects of body mass index, gender, age, social support, and time of assessment. In the second step of the analysis, we added participants’ attachment avoidance and anxiety scores as predictors. The analysis revealed that older age corresponded with marginally higher fasting basal glucose, *b* = 0.11, 95% CI for *b* (–0.01, 0.23), β = 0.11, *p* = 0.067. The addition of attachment scores in the second step of the analysis significantly increased the amount of variance accounted for, *ΔF*(2, 257) = 4.76, *p* = 0.009, *ΔR*^2^ = 0.04. Consistent with our hypothesis, the analysis revealed that the higher the participants’ attachment avoidance score, the greater their fasting basal glucose level, *b* = 2.18, 95% CI for *b* (0.74, 3.62), β = 0.22, *p* = 0.003, replicating the results of Study 1. Attachment anxiety was not related to participants’ fasting basal glucose, *b* = –0.74, 95% CI for *b* (–2.19, 0.71), β = –0.07, *p* = 0.32. Supplementary logistic regression analyses revealed that attachment avoidance was not significantly related to the likelihood of suffering from diabetes, *Exp(b)* = 1.11, *p* = 0.72, or obesity, *Exp(b)* = 0.86, *p* = 0.37, indicating that attachment avoidance was associated with normal levels of fasting basal glucose.

**FIGURE 2:**
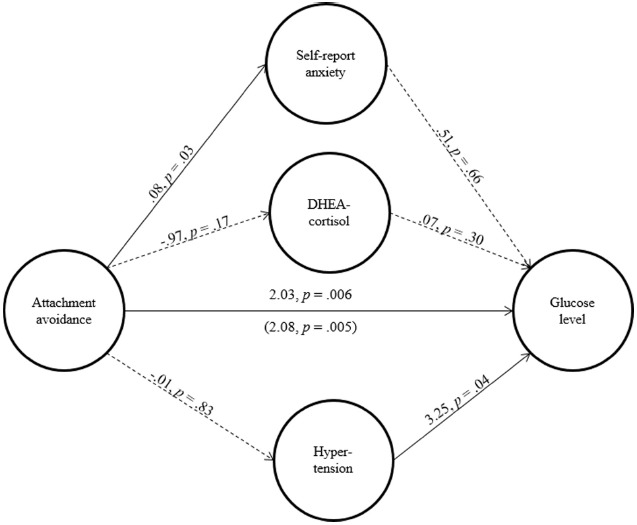
**The structural model above depicts a multiple mediation model to examine whether tension and stress-related indicators mediate the link between attachment avoidance and fasting basal glucose level.** Solid paths represent significant predictions; dotted paths represent non-significant predictions. The coefficient in parenthesis represents the association between attachment avoidance and glucose level after the inclusion of the tension and stress-related indicators.

To assess the possibility that avoidance from social ties relates to greater fasting basal glucose level through heightened distress and tension, we conducted a multiple mediation analysis (Preacher and Hayes, 2008), where the association between attachment avoidance and fasting basal glucose level was modeled as being mediated by three indicators of tension and distress: (a) self-report level of anxiety, (b) cortisol/DHEA ratio, and (c) endorsement of clinical hypertension (see Figure 2). Following Wang and Dvorak (2010), we also adjusted the analysis for the possible effects of body mass index, gender, age, social support, and time of assessment. The specified mediation pathways did not account for the association between attachment avoidance and fasting basal glucose level (i.e., bias-corrected bootstrap analyses were not significant, which indicate non-significant mediation pathways). Also, the association between attachment avoidance and basal glucose level remained significant after the inclusion of all three indicators of tension and distress, *b* = 2.08, *p* = 0.005. Given that we had sufficient power (i.e., above 80%) to discover weak-to-moderate mediation paths (i.e., mediation paths comprised two βs of 0.20; Fritz and MacKinnon, 2007), these null results are unlikely to stem from insufficient statistical power.

In line with our prediction, people high in attachment avoidance tended to maintain higher fasting basal glucose levels than their more secure counterparts. In addition, elevated tension and stress did not account for this association. Studies 1 and 2 have only linked attachment avoidance with higher basal glucose level but not with greater consumption of sugar-rich food. In addition, Studies 1 and 2 are correlational in nature and preclude the ability to draw conclusions regarding the possible causal link between avoidance and glucose-related indices. We designed Study 3 to address these limitations.

## Study 3

In Study 3, we examined the link between trait-like attachment avoidance, manipulation of an asocial state, and consumption of sugar-rich food. We predicted that the higher the attachment avoidance, the greater the consumption of sugar-rich food. We also predicted that under a state of asocial expectations, people would consume more sugar-rich food than under a control condition.

### Materials and Methods

#### Participants

One-hundred-and-eight Israeli participants (59 women and 49 men), ranging in age from 17 to 30 years (*Mdn* = 22), volunteered to participate in the study. Study 3 was approved by the Interdisciplinary Center (IDC) Herzliya’s IRB.

#### Measures and Procedure

The participants were a convenience sample recruited in IDC. Participants were approached by a research assistant between 11 and 12 AM, who asked them to participate in a study on storytelling appraisal. Upon their consent, participants were invited to a quiet room, and were asked to complete self-report measures of attachment orientation. Specifically, attachment orientation was assessed with a Hebrew version of the ECR scales (Brennan et al., 1998). Participants rated the extent to which each item was descriptive of their feelings in close relationships on a 7-point scale ranging from *not at all* (1) to *very much* (7). Eighteen items assessed attachment anxiety (e.g., “I worry about being abandoned”) and 18 assessed avoidance (e.g., “I prefer not to show a partner how I feel deep down”). The reliability and validity of these scales have been repeatedly demonstrated (Mikulincer and Shaver, 2007). In our study, Cronbach αs were 0.70 for the anxiety items and 0.92 for the avoidance items. Mean scores were computed for each scale, and the two scores were significantly correlated, *r*(106) = 0.55, *p* < 0.001.

After completing the questionnaire, each participant was presented with one randomly assigned hypothetical story, out of two possible stories. The stories were used to manipulate a state of asocial expectations. Participants were asked to read and appraise the story by writing about their thoughts and feelings following the story that they have just read. In the *asocial* condition participants read the following paragraph: “Don doesn’t like to come home from college for the holidays. If he could afford to go someplace else, he would. He doesn’t have many friends back home but he likes it that way. He is not very open with members of his family, and he has few friends he can depend on and talk to when he is in need of support. He prefers to be self-reliant”.

In the *control* condition participants read the following paragraph: “Don, a college student, runs errands for a living. In his regular route, he first goes to the supermarket to buy groceries for his customers for the week. Next, he stops by the mechanic’s shop to change the oil for a customer’s car. He then goes to pick up tickets to a basketball game for another customer. He gets himself tickets for him and his friends as well. After dropping off all the tickets, he heads over to the game. Then, after the game, he heads back to campus and finishes readings and assignments he has for class the next day.”

Participants then completed a short demographic sheet, were thanked, and were offered to eat as much Elite^™^ chocolate chunks as they like as a compensation for their time. The number of chocolate chunks eaten served as the dependent variable.

### Results and Discussion

**FIGURE 3:**
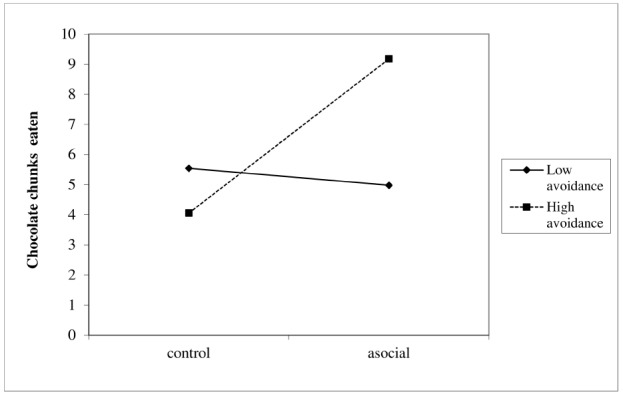
**Probing the interaction between attachment avoidance and condition in predicting the number of chocolate chunks eaten.** Only among participants high in attachment avoidance, the manipulation of asocial tendencies generated an increase in the amount of chocolate chunks eaten.

The number of chocolate chunks eaten was examined using hierarchical regression. In the first step of the analysis, we introduced the measures of attachment avoidance and anxiety, and condition (–1 = control, 1 = asocial) as predictors. We also added gender as a covariate. In the second step of the analysis, we added the interactions between attachment avoidance and condition, and attachment anxiety and condition. To ease interpretation of results and to avoid multicollinearity, we centered the measures of attachment avoidance and anxiety around their grand mean. Consistent with our hypothesis, the analysis revealed that the higher participants’ attachment avoidance, the more chocolate chunks they ate, *b* = 1.22, 95% CI for *b* (0.01, 2.43), β = 0.23, *p* = 0.05. Participants in the asocial condition ate more chocolate chunks than participants in the control condition, *b* = 1.26, 95% CI for *b* (0.39, 2.13), β = 0.27, *p* = 0.005. These effects, however, were moderated by the interaction between attachment avoidance and condition, *b* = 1.42, 95% CI for *b* (0.15, 2.69), β = 0.27, *p* = 0.029. Using Hayes’s (2013) procedure, which is based on simple slopes test, we found that among participants high in attachment avoidance, the manipulation of asocial expectations generated an increase in the amount of chocolate chunks eaten, *b* = 2.56, *p* < 0.001 (see Figure 3). Among people low on attachment avoidance, and, hence, the more secure, the effect of the asocial manipulation was not significant, *b* = 0.28. Finally, the analysis indicated that attachment anxiety was associated with fewer chocolate chunks eaten, *b* = –1.45, 95% CI for *b* (–2.57, –0.33), β = –0.30, *p* = 0.01.

In line with our prediction, people high in attachment avoidance tended to eat more chocolate chunks especially after being primed with asocial sentiments, while secure people, who commonly trust others and work in collaboration with them, did not.

## Study 4

In Study 3, we have found that manipulating a state of asocial expectations affected the consumption of sugar-rich food among people high in attachment avoidance but not among secure people. We designed Study 4 to examine whether people who score high in attachment avoidance may consume more sugary cereal after being paired with another person under stressful conditions. Participants underwent a challenging task either alone or in pairs, and then ate cereals high in sugar for breakfast. We predicted that attachment avoidance will be linked with greater consumption of sugar-rich food specifically in the paired condition, because asocial people do not expect to benefit from the aid of others as compared with more secure individuals.

### Materials and Methods

#### Participants

One-hundred-and-fifteen Israeli undergraduate students (65 women and 50 men), ranging in age from 18 to 33 years (*Mdn* = 22), from the IDC, Herzliya participated in the study for course credit. Study 4 was approved by the IDC Herzliya’s IRB.

#### Measures and Procedure

Participants were invited to IDC’s laboratory complex between 11 and 12 AM either alone or in pairs. They were told that in order to save them time and effort, they will be taking part in two unrelated studies. First, participants were told they are going to take part in a study on body temperature and later in another experiment on eating habits. The procedure was a cover story that was used to conceal the true nature of our study. After giving their informed consent, participants were asked to complete two questionnaires. Attachment orientation was assessed with a Hebrew version of the ECR, as in Study 3. In our study, Cronbach αs were 0.76 for the anxiety items and 0.89 for the avoidance items. Mean scores were computed for each scale, and the two scores were significantly correlated, *r*(113) = 0.41, *p* < 0.001. Next, to support our cover story, participants completed a bogus body temperature questionnaire, which consisted of rating the frequency of daily habits related to temperature (e.g., “I like showering in very hot water”), on a 7-point scale ranging from *disagree strongly* (1) to *agree strongly* (7).

After completing these questionnaires, participants were put through a cold presser stress task (Lovallo, 1975). It is an extensively used laboratory stress manipulation, in which participants are put through a cold stimulus for few minutes. In this case, participants were asked to put their hands in ice water up to a minute and a half. Participants in the paired condition each had their own bowl but sat in close contact with each other.

Following the task, participants were thanked and asked to take part in our second study, which explored the link between daily habits and appetite. They filled in a bogus habits questionnaire, which consisted of rating different everyday habits. Next, they were given a box of cereal and a bowl, and were asked to pour in the bowl as much cereal as they think they might eat for breakfast. Participants in the paired condition were asked to do this separately, having one participant wait in a second room while the first participant poured their cereal. The bowl was then put aside, and the participants were debriefed and thanked. Once the participants left the bowls were weighed by EatSmart^™^ Precision Pro scale.

### Results and Discussion

**FIGURE 4:**
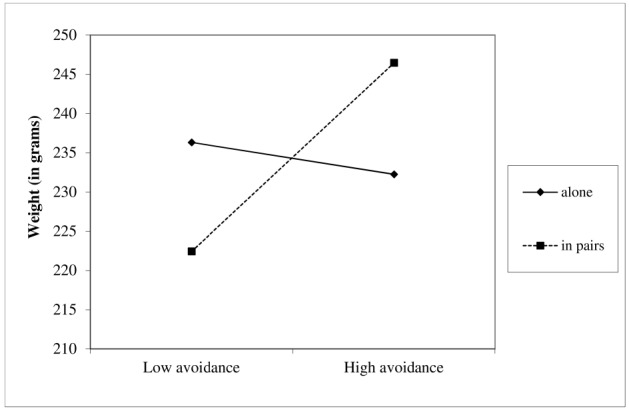
**Probing the interaction between attachment avoidance and condition in predicting the weight of cereals.** Only in the paired condition, the higher the attachment avoidance, the greater the amount of cereals allocated.

The weight of cereals was examined using hierarchical regression. In the first step of the analysis, we introduced the measures of attachment avoidance and anxiety, and condition (–1 = alone, 1 = in pairs) as predictors. We also added gender as a covariate. In the second step of the analysis, we added the interactions between attachment avoidance and condition, and attachment anxiety and condition. To ease interpretation of results and to avoid multicollinearity, we centered the measures of attachment avoidance and anxiety around their grand mean. Consistent with our hypothesis, the analysis revealed a significant interaction between attachment avoidance and condition, *b* = 7.02, 95% CI for *b* (0.70, 13.35), β = 0.23, *p* = 0.03. Hayes’s (2013) procedure indicated that only in the paired condition, higher attachment avoidance corresponded with a higher quantity of cereal, *b* = 12.02, *p* < 0.001 (see Figure 4). In the alone condition, the link between attachment avoidance and weight of cereals was not significant, *b* = 2.02.

In line with our prediction, attachment avoidance was linked with greater allocation of sugar-rich food specifically in the paired condition. It seems that secure individuals can benefit from the presence of others while performing a stressful task. As a result, they are less exhausted and/or expect to need a lower amount of metabolic resources at the aftermath of the task. Avoidant people share no such expectation, and hence, they may be more exhausted and/or expect to need a greater amount of metabolic resources as compared with secure people.

## General Discussion

Economy of action—a principal of biological systems—posits that all organisms must take in more energy than they expend if they are to survive, grow, and reproduce (Proffitt, 2006; Gross and Proffitt, 2013). In practice, the economy of action principle creates pressure for all organisms to conserve resources whenever possible. Recently, Coan and colleagues (Beckes and Coan, 2011; Coan and Sbarra, 2015) proposed that socially oriented people conserve, on average and in the aggregate, more energy than asocial people, because they construe—in largely implicit ways—social relationships as opportunities to conserve resources by sharing the load of life’s myriad situational demands (Coan et al., 2006). In the studies reported here, we have examined the possibility that people who tend to avoid social resources would devote higher levels of a rapidly deployable metabolic resource—glucose—to their bloodstream and to consume more sugar-rich food, in order to increase their all-purpose access to that resource. That is because they are not disposed to share the cost of many of life’s metabolically expensive activities, from physical labor to complex decision-making, problem solving and vigilance for threats. Specifically, we hypothesized that people high in attachment avoidance would maintain higher basal levels of glucose—the predominant organic fuel for a multitude of biosynthetic processes, including cerebral metabolism (Vannucci and Vannucci, 2000)—and consume more sugary foods.

Results supported these hypotheses. In Study 1, we found that women who chronically tend to distance themselves from social resources—those high in attachment avoidance—maintained higher fasting basal glucose levels than more socially oriented women. In Study 2, we replicated the results of Study 1 in a different culture, among women as well as men, from a different age group, and using a different measure of attachment avoidance. Thus, the association between social avoidance and basal glucose level seems robust. Study 2 also showed that although people high in attachment avoidance maintain higher basal levels of glucose, there was no evidence that they are exceptionally prone to glucose-related disorders such as diabetes and obesity.

One advantage of Study 2 was that it allowed us to address alternative explanation for the association between the avoidance of social resources and fasting basal glucose levels. For example, research suggests that a small and unsupportive social network, and especially feelings of loneliness, may trigger severe feelings of negativity as well as compromised health (Cacioppo et al., 2003) via mechanisms that include—among other things—elevated basal glucose levels (Armario et al., 1996; Whisman, 2010). Thus, the high amount of basal glucose associated with attachment avoidance may stem merely from the stress of relative social isolation and not because of a “bet” that more personal resources are needed because social resources are unlikely to obtain. Our results indicated, however, that the association between attachment avoidance and fasting basal glucose level remained significant even after statistically adjusting for three sensitive and multimodal indicators of distress: self-reported anxiety, symptoms of hypertension, and the cortisol/DHEA ratio. Thus, the association between attachment avoidance and basal glucose results from something other than current distress in our participants. In addition, the effect of attachment avoidance remained significant after adjusting for various other measures that might affect the pattern of results, including age, gender, body mass, social support, and time of assessment. We posit again that the association between fasting glucose and attachment avoidance reflects a “bet” by more avoidant participants that the challenges they face will require independent solutions, a bet that itself probably stems from a social history of unreliable or deficient access to social resources (Bowlby, 1973; Coan, 2008). Our contention is in line with recent findings linking glucose-related decision making with Bayesian reasoning (Dickinson et al., 2014).

In Study 3, we found that people who chronically tend to distance themselves from social resources—those high in attachment avoidance—consume more sugar-rich food, especially when reminded of asocial tendencies. Study 4 extended these findings, suggesting that after facing a stressful task in the presence of others, avoidant people gather more sugar-rich food than more socially oriented people. These results are in keeping with recent research linking loneliness and lack of social network with greater consumption of sugar-rich beverages (Henriksen et al., 2014).

Our findings are also in line with recent theory and research regarding the potentially adaptive nature of social avoidance. Specifically, Ein-Dor et al. (2010) contended that although avoidant people are more likely to rely on self-protective fight-or-flight responses in times of danger, they might also more rapidly identify and enact protective maneuvers when faced with a threatening situation—an advantage contributing to the relative frequency of the trait. At the individual level, the potential adaptability of this approach is obvious, but this kind of behavior may sometimes save other people’s lives as well, by thwarting a threat or identifying an escape route that can benefit the group to which the avoidant person belongs. Indeed, Ein-Dor et al. (2011b) did observe that attachment-related avoidance was associated with speedier escape responses to an experimentally manipulated danger—a room gradually filling with smoke, apparently because of a malfunctioning computer—and therefore with greater group safety. In addition, Ein-Dor et al. (2012) observed that avoidant individuals were better equipped than their less avoidant peers to succeed and be satisfied with professional singles tennis and computer science because these fields reward self-reliance, independence, and the ability to work without proximal social support from loved ones. Thus, it seems that trait-like attachment avoidance is more of an adaptation to a relatively independent way of life, a view that is somewhat different that the contemporary view of avoidant individuals as more globally deficient (Mikulincer and Shaver, 2007).

The results of this research also add to a growing body of evidence for the adaptive nature of individual variation in personality. For instance, Nettle (2006) has argued that such variability can be understood in terms of tradeoffs among fitness costs and benefits: “Behavioral alternatives can be considered as tradeoffs, with a particular trait producing not unalloyed advantage but a mixture of costs and benefits such that the optimal value for fitness may depend on very specific local circumstances” (p. 625).

There are, of course, some limitations to our studies. First, we emphasize that the correlational nature of Studies 1 and 2 precludes confident conclusions about the direction of causality in the link between avoidance and fasting basal glucose levels. Theory and research on attachment, however, do suggest that attachment orientations, including attachment avoidance, are formed in early childhood and are moderately stable over periods of years (Mikulincer and Shaver, 2007). Moreover, Coan and colleagues (Coan, 2008, 2010; Beckes and Coan, 2011; Coan and Sbarra, 2015) have drawn from a large body of animal and human neuroscientific research to specifically predict that relative isolation should cause increased demands on metabolic resources. In addition, Studies 3 and 4 have revealed that manipulating asocial tendencies and/or people’s social network induces changes in people’s consumption of sugar-rich food. In combination, there is reason to believe that avoidance is driving the association with fasting basal glucose levels and not vice versa. Future studies might also benefit from the inclusion of other personality measures to rule out the possibility that our findings regarding avoidance are attributable to other traits. Of course, by now many attachment studies have included measures of the Big Five personality traits, and attachment measures typically predict theoretically expected variables even when the Big Five traits are statistically adjusted for (Mikulincer and Shaver, 2007).

Ultimately, our findings raise the possibility that people who consistently avoid the use of social resources and strive to maintain independence, compensate for these tendencies in part by maintaining a higher basal glucose level in their blood, and possibly by consuming more sugar-rich food—a strategy for rapidly accessing the metabolic fuel that helps them successfully face various life challenges alone.

### Conflict of Interest Statement

The authors declare that the research was conducted in the absence of any commercial or financial relationships that could be construed as a potential conflict of interest.

## Footnotes

1 Curve estimation regression analysis revealed no indication of quadratic relations between attachment avoidance and fasting basal glucose level, *t*(258) = 0.23, *p* = 0.82. Thus, in subsequent analyses we used linear-based regression analyses.
